# Altered serum human cytomegalovirus microRNA levels are common and closely associated with the inflammatory status in patients with fever

**DOI:** 10.3389/fimmu.2022.1079259

**Published:** 2022-12-14

**Authors:** Cheng Wang, Yunhua Zhu, Penglu Chen, Chen Wang, Wanqing Zhou, Cuiping Zhang, Jing Wang, Xi Chen, Meng Ding, Chunni Zhang, Jun-Jun Wang, Chen-Yu Zhang

**Affiliations:** ^1^ Department of Clinical Laboratory, Jinling Hospital, State Key Laboratory of Pharmaceutical Biotechnology, NJU Advanced Institute for Life Sciences (NAILS), School of Life Sciences, Nanjing University, Nanjing, China; ^2^ Nanjing Drum Tower Hospital Center of Molecular Diagnostic and Therapy, Chinese Academy of Medical Sciences Research Unit of Extracellular RNA, State Key Laboratory of Pharmaceutical Biotechnology, Jiangsu Engineering Research Center for MicroRNA Biology and Biotechnology, NJU Advanced Institute of Life Sciences (NAILS), Institute of Artificial Intelligence Biomedicine, School of Life Sciences, Nanjing University, Nanjing, China

**Keywords:** HCMV, human cytomegalovirus, serum, fever, risk factor, immune disorders, microRNA

## Abstract

**Background:**

Fever has a complicated etiology, and diagnosing its causative factor is clinically challenging. Human cytomegalovirus (HCMV) infection causes various diseases. However, the clinical relevance, prevalence, and significance of HCMV microRNAs (miRNA) in association with fever remain unclear. In the present study, we analyzed the HCMV miRNA expression pattern in the serum of patients with fever and evaluate its clinical associations with occult HCMV infection status in immune disorders.

**Methods:**

We included serum samples from 138 patients with fever and 151 age-gender-matched controls in this study. First, the serum levels of 24 HCMV miRNAs were determined using a hydrolysis probe-based stem-loop quantitative reverse transcription polymerase chain reaction (RT-qPCR) assay in the training set. The markedly altered miRNAs were verified in the validation and testing sets. The serum HCMV IgG/IgM and DNA titers in the testing cohort were also assessed using enzyme-linked immunosorbent assay (ELISA) and RT-qPCR, respectively.

**Results:**

The majority of HCMV miRNAs were markedly upregulated in the serum of fever patients. We selected the five most significantly altered HCMV miRNAs: hcmv-miR-US4-3p, hcmv-miR-US29-3p, hcmv-miR-US5-2-3p, hcmv-miR-UL112-3p, and hcmv-miR-US33-3p for validation. These miRNAs were also significantly elevated in the serum of fever patients in the validation and testing sets compared with the controls. Logistic regression analysis revealed that the five miRNAs were novel potential risk factors for fever. Notably, the serum levels of four of the five confirmed HCMV miRNAs were significantly associated with blood C-reaction protein concentrations. Moreover, the five HCMV miRNA levels were closely correlated with the HCMV DNA titers in the testing cohort.

**Conclusion:**

HCMV infection and activation are common in fever patients and could be novel risk factors for fever. These differentially expressed HCMV miRNAs could enable HCMV activation status monitoring in immune disorders.

## 1 Introduction

Fever, including undifferentiated fever and fever of unknown origin (FUO), is frequently observed in patients seeking health care in China. However, the complex etiology of fever and its delayed diagnosis complicates the treatment (1–3). Recent epidemiological surveys have demonstrated that fever is prevalent in both developing and developed countries, and the causes of fever vary depending on the region and time (4). Nowadays, the most frequently reported etiologies of fever include infection, non-infectious inflammatory disease (NIID), and malignancy. However, these factors cause fever in about 60–80% of fever patients, and many patients remain undiagnosed (5, 6). The most frequently used diagnostic procedures for fever are imaging methods, blood tests, blood cultures, and molecular detection (7). Blood cultures and serum procalcitonin tests can discriminate fever resulting from bacterial infection from other fevers, but the unfulfilled positive rate and time-consuming procedures often obscure and delay the diagnosis of fever (8). Thus, accurate diagnosis and timely management of fever remain a challenge for physicians in clinical therapy.

Reportedly, human herpes virus (HHV) infection is an important etiological agent for fever, especially for FUO (9). The study highlights an association between HHV infection and the clinical signs of fever, providing direct evidence that HHV cell-free nucleic acids detected in circulation might help in the diagnosis and treatment of fever. As one of the largest and most complex members of the human beta herpes virus family, human cytomegalovirus (HCMV) is a ubiquitous human pathogen. The seroprevalence data revealed that > 90% of people have been infected with HCMV, and the infection risk increases with age. Once infected, the infection persists lifelong and may undergo periodic reactivation (10). HCMV infection and activation can cause congenital birth defects and morbidity in immunocompromised patients and allogeneic bone marrow or organ-transplant recipients. However, the molecular mechanism by which HCMV infects the host, maintains latency, and reactivates remains unclear (10). Patients with fever frequently present with immunocompromised conditions or immunodepression during the disease. However, the clinical association between HCMV and fever has rarely been explored. Moreover, the high prevalence of HCMV IgG and a short window period for HCMV IgM detection in blood limits HCMV infection and reactivation surveillance.

MicroRNAs are small noncoding RNA molecules that bind to the 3′ untranslated region (3′-UTR) of a target messenger RNA and repress its translation, thus controlling protein expression at the post-transcriptional level in virtually all eukaryotic organisms and some virus, including HCMV (11). HCMV encodes 26 known mature miRNAs. Some of the HCMV-encoded miRNAs target both viral and host genes, including important immune modulators. Furthermore, infections with HCMV can alter the expression of host miRNAs to benefit virus replication (11, 12). Human body fluids also contain stable miRNAs, and dysregulated serum miRNAs are blood-based biomarkers for various diseases, such as cancer, diabetes, and infectious diseases (13). These pieces of evidence imply that expression profiles of altered host miRNAs combined with HCMV miRNAs in the serum might serve as potential diagnostic tools for HCMV-related diseases and be involved in disease occurrence and progression. A pioneering study reported that a circulating miRNA profile comprising two host miRNAs and one HCMV miRNA, hcmv-miR-UL112, is a novel noninvasive biomarker for essential hypertension (14). Subsequently, another group found the same HCMV miRNA (hcmv-miR-UL112) more prevalent in the serum of diabetes mellitus and glioblastoma multiform patients as compared with rheumatoid arthritis patients and healthy subjects (15). Furthermore, our group also found that dysregulated HCMV miRNAs in circulation were closely associated with oral lichen planus, adverse pregnancy outcomes, and treatment efficacy of chronic hepatitis B patients (16–18). Additionally, circulating HCMV miRNAs could be encapsulated by extracellular vesicles and participate in immune disorders (16, 19, 20).

Currently, despite the high infection rates of HCMV in human beings, little is known about the HCMV-encoded miRNA patterns in the serum of fever patients with immune disorders. The clinical relevance of HCMV miRNAs in fever also remains incompletely characterized. Thus, in the present study, we used hydrolysis probe-based reverse transcription quantitative real-time PCR (RT-qPCR) to examine the comprehensive expression profile of serum HCMV miRNAs in a cohort of fever patients to analyze the relations between HCMV and fever. We also aimed to evaluate the possible clinical significance and associated pathogenesis of serum HCMV miRNAs, which could provide useful information for fever management.

## 2 Materials and methods

### 2.1 Study population, sample collection, and study design

The present study complies with the World Medical Association Declaration of Helsinki and is approved by the ethics committee board of Jinling hospital. Written informed consent was obtained from all participants. For the patients who had a compromised capacity to consent, written informed consent was obtained from the legally authorized representative on the behalf of the participant. A total of 138 fever patients who were treated at the Department of Integration of Traditional Chinese and Western Medicine of Jinling Hospital (Nanjing, China) between November 2013 and November 2016 were enrolled in this study. The inclusion criterion in the study was: patients with a fever (≥ 37.3 °C) with no apparent diagnosis after an initial outpatient or hospital evaluation, including a careful history, physical examination, and initial laboratory assessment. Patient details, including demographic characteristics, medical history, blood parameters, blood cultures (if performed), imaging and endoscopy (if performed), final diagnostic, and outcome data were collected and retrospectively analyzed. In addition, 151 age-gender-matched individuals from the Healthy Physical Examination Centre of Jinling Hospital showing no evidence of fever and/or other abnormalities were selected as normal controls.

**Figure 1:**
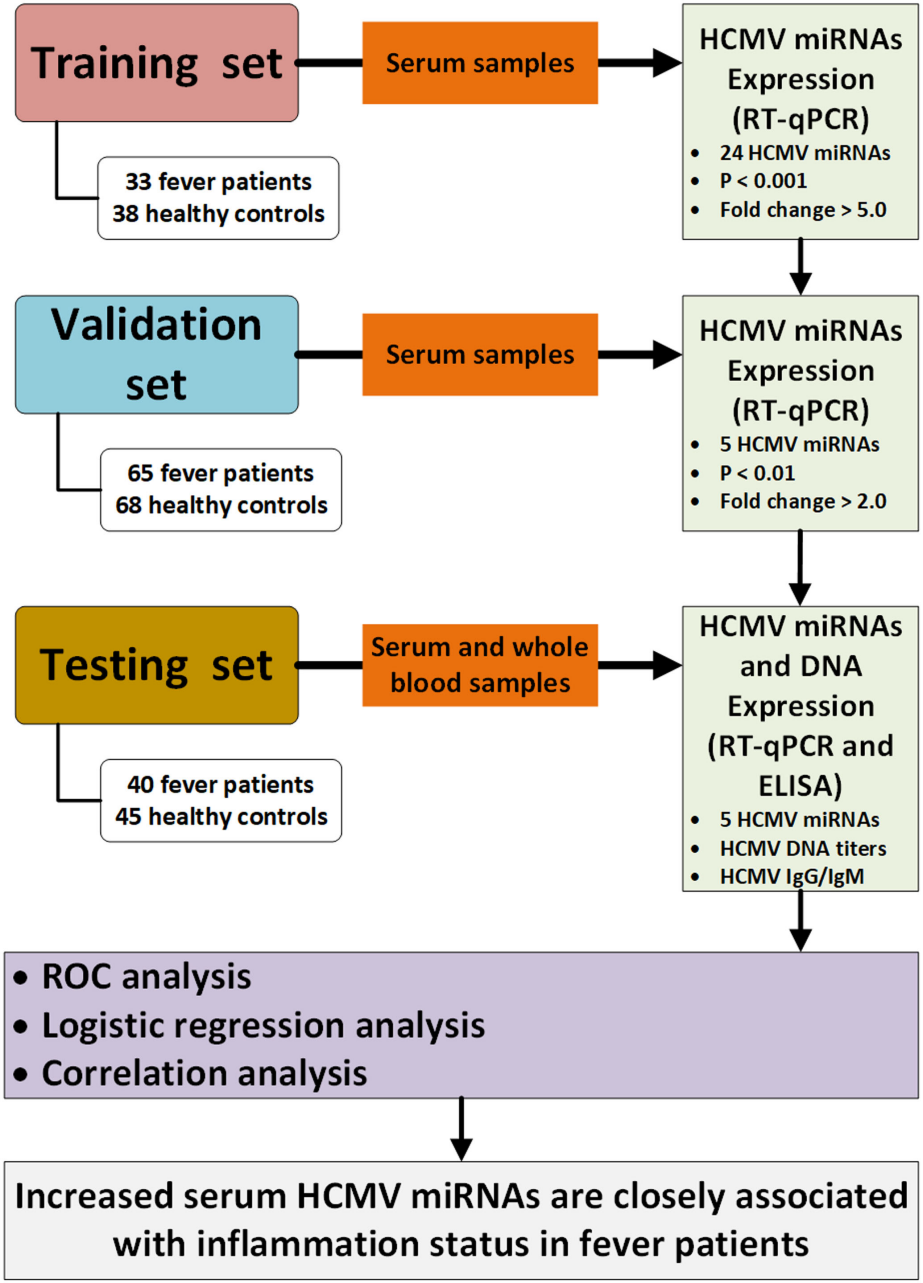
Flow chart of the experimental design. HCMV, human cytomegalovirus; Cq, quantification cycle; RT-qPCR, quantitative reverse transcription polymerase chain reaction; ELISA, enzyme-linked immunosorbent assay; ROC, receiver operating characteristic.

A multiphase case-control study was designed to explore the dysregulated HCMV miRNA profile in the serum of fever patients. We initially screened the 24 HCMV miRNAs and their expression patterns in a cohort (the training set) constituting 33 patients and 38 healthy controls. Subsequently, the most markedly altered HCMV miRNAs were further confirmed in another cohort (the validation set) containing 65 patients and 68 controls. Finally, the selected miRNAs were examined in a cohort (the testing set), including 40 patients and 45 controls. Moreover, we also measured the peripheral blood HCMV DNA titers and serum anti-HCMV IgG/IgM content in the testing set. A schematic representation of the study design and workflow is shown in **Figure 1**.

### 2.2 Serum RNA isolation and RT-qPCR assay of HCMV-encoded miRNAs

Total RNA was extracted from 100 μL of serum using a one-step phenol/chloroform purification method and precipitated using isopropyl alcohol as previously described (21). Hydrolysis probe-based RT-qPCR was carried out using a TaqMan miRNA PCR kit (Applied Biosystems, Foster City, CA, USA) according to the manufacturer’s instructions with a minor modification as previously described (21). Briefly, 2 μL of total RNA was reverse transcribed to cDNA using AMV reverse transcriptase (TaKaRa, Dalian, China) and the stem-loop RT primer (Applied Biosystems, Foster City, CA, USA). Real-time PCR was performed using hydrolysis miRNA probes on a Roche LightCycler^®^ 480 II PCR System (Roche Diagnostics, Mannheim, Germany). All reactions, including no-template controls, were performed in triplicates. The quantification cycle (Cq) values were determined using the fixed threshold settings. To control the variability in RNA extraction and purification procedures, an exogenous plant small molecular RNA, MIR2911 (5′-GGCCGGGGGACGGGCUGGGA-3′), was spiked into each sample with a final concentration of 10^6^ fmol/L during RNA isolation as a synthetic external reference for the normalization of serum miRNAs. Relative levels of HCMV miRNAs were then normalized to the exogenous MIR2911 and calculated using the comparative Cq method (2^-ΔCq^).

### 2.3 HCMV titers, anti-HCMV IgG, and IgM antibodies determination

We examined the copy number of HCMV DNA using qRT-PCR in the peripheral blood samples of fever patients and controls in the testing set as previously described (14, 16). Briefly, the sequences of the sense and antisense primers for HCMV DNA quantification were 5′-CACGGTCCCGGTTTAGCA-3′ and 5′-CGTAACGTGGACCTGACGTTT-3′. The probe sequence was 5′-TATCTGCCCGAGGATCGCGGTTACA-3′. The 5′ and 3′ ends of the probe were labeled with FAM and TAMRA dyes, respectively. HCMV DNA was extracted from the peripheral blood leukocytes (PBLs) using the QIAamp Min Elute Virus Spin (Qiagen, Hilden, Germany) and QIAamp DNA Mini kit (Qiagen, Hilden, Germany) according to the manufacturer’s protocol. We tested 20 μL of each DNA extract with TaqMan PCR assays on a 96-well plate for < 45 cycles. A ten-fold diluted recombinant plasmid containing the HCMV target sequence was used as a template for standard curve preparation.

ELISA tests for anti-HCMV IgG and IgM antibodies in serum were performed with a commercially available ELISA kit (MEDSON, NJ, USA) according to the manufacturer’s instructions. Briefly, for anti-HCMV IgG testing, the absorbance of serum anti-HCMV IgG was assessed using the ELISA kit, and its absolute concentrations were calculated from the calibration curve. For anti-HCMV IgM testing, ELISA values of < 1.0, 1.0–1.2, and > 1.2 were considered as a negative result, an equivocal infection, and a positive result, indicating prior exposure to HCMV (for anti-HCMVIgG) or acute infection with HCMV (for anti-HCMV IgM), respectively.

### 2.4 Statistical analysis

Statistical analysis was performed with GraphPad Prism 9.0 software or SPSS statistical software (version 23.0). The miRNA levels are represented as means and standard errors (Mean ± SEM) and other clinical variables as mean ± SD. The data analyses were performed using the non-parametric Mann–Whitney tests or Kruskal-Wallis test with statistical significance set at *P* < 0.05. Forward stepwise univariate and multivariate logistic regression analyzes were conducted to evaluate the influences of serum HCMV miRNAs on fever patients. ROC curves were constructed, and the areas under the ROC curves (AUCs) were calculated to evaluate the discrimination ability of serum HCMV miRNA for fever prediction. Spearman rank correlation analysis was used to analyze the relationship between the miRNA concentrations and clinical features or other parameters.

## 3 Results

### 3.1 Study population and final diagnosis

The present study enrolled 138 fever patients, including 87 (63%) males and 51 (37%) females, with a mean age of 58.00 ± 23.57 years (**Table 1**). Based on the medical records, 122 (88.4%) patients ultimately had their underlying disease diagnosed, of which 87 (63%) had infectious diseases, 20 (14.5%) had non-infectious connective tissue diseases, four (2.9%) had hematological malignancies, 11 (8.0%) had non-infectious inflammatory diseases, and 16 (11.6%) remained undiagnosed. As shown in **Table 1**, pulmonary infection, upper respiratory tract infection, vasculitis, and connective tissue diseases were the most common diagnoses. Laboratory test results, including hematological index and serological and immunological parameters (if performed), are also listed in **Table 1**.

**Table 1:** Demographic and clinical information of the fever patients and healthy controls^a^.

Variables		Control	Fever	P value
	(n = 151)	(n = 138)	
**Age (years)**		53.87 ± 10.44	58.00 ± 23.57	0.052^b^
**Sex**				0.799^c^
	Male	93	87	
	Female	58	51	
**Hematological index**				
	WBC (× 10 ^9^/L)	5.79 ± 1.50	8.48 ± 5.43	< 0.0001^d^
	Lymphocyte (%)	35.33 ± 8.05	21.99 ± 14.12	< 0.0001^d^
	Monocytes (%)	5.80 ± 1.53	7.93 ± 3.98	< 0.0001^d^
	Neutrophil (%)	55.79 ± 8.98	68.01 ± 15.82	< 0.0001^d^
	Acidophilicgranulocyte (%)	2.47 ± 1.71	1.76 ± 2.65	< 0.0001^d^
	Basophil (%)	0.41 ± 0.23	0.32 ± 0.35	< 0.0001^d^
**C-reactive protein (mg/L)**		< 0.50	48.16 ± 50.42	
**Procalcitonin (μg/L)**			2.03 ± 7.92	
**Interleukin-6 (ng/L)**			44.18 ± 49.07	
**Final diagnosis for fever**				
	Infection			87 (63 %)
			Tuberculosis	1
			Bacteremia	2
			Brucellosis	1
			Sepsis	3
			Pulmonary infection	34
			Mycoplasma pneumonia	2
			Upper respiratory tract infection	32
			Urinary tract infection	4
			Intracranial infection	1
			Infective endocarditis	2
			Gastrointestinal tract infection	1
			Monocytic angina	2
			Influenza viruses	1
			Herpes	1
	Autoimmunological disease			20 (14.5 %)
			Vasculitis	7
			Adult still disease	1
			Sjogren syndrome	1
			Systemic lupus erythematosus	2
			Overlap syndrome	1
			Undifferentiated connective tissue disease	6
			Erythema nodosum	2
	Hematological malignancies			4 (2.9 %)
			Lymphoma	3
			Malignant plasma cell dyscrasia	1
	Non-infectious inflammatory diseases			11 (8.0 %)
			Necrotizing lymphadenitis	4
			Thyroiditis	1
			Other	6
	Undiagnosed			16 (11.6 %)

^a^Data are mean (SD) or number (%). ^b^Compared with control group. ^c^Two sides χ^2^ test. ^d^P values were calculated by the Mann-Whitney U test.

### 3.2 Expression profiles of HCMV-encoded miRNAs in the serum of fever patients

To fully characterize the serum HCMV miRNA expression signature in fever patients, we measured the expression pattern of 24 HCMV miRNAs (miRBase 22.0) in 33 randomly assigned fever patients and 38 matched controls (referred to as the training set) using RT-qPCR analysis. The RT-qPCR results showed that HCMV miRNAs were readily detected in both fever patients and controls. Of the 24 HCMV miRNAs examined, 17 miRNAs were significantly upregulated in the serum of fever patients compared with the controls (*P* < 0.05). The relative expression levels of the 24 HCMV-encoded miRNAs in fever patients and controls are shown in **Supplemental Data Table 1**.

Of the 17 markedly altered miRNAs, five with a high elevation (fold change > 5 and *P* < 0.001), including hcmv-miR-US4-3p, hcmv-miR-US29-3p, hcmv-miR-US5-2-3p, hcmv-miR-UL112-3p, and hcmv-miR-US33-3p, were then selected for further verification (**Table 2**). These miRNAs are reported to be related to CMV latency, reactivation, and immune evasion.

**Table 2:** Serum levels of the markedly altered HCMV-encoded miRNAs in fever patients arranged into the training and validation sets^a^.

HCMV miRNA	Control	Fever	P value	Fold change
**Training set**	**(n = 38)**	**(n = 33)**		
hcmv-miR-US4-3p	29.87 ± 5.55	209.7 ± 61.02	< 0.0001	7.02
hcmv-miR-US29-3p	6.56 ± 1.52	34.12 ± 9.72	< 0.0001	5.2
hcmv-miR-US5-2-3p	2.74 ± 0.60	14.17 ± 4.07	0.0001	5.17
hcmv-miR-UL112-3p	123.3 ± 15.92	867.7 ± 312.0	0.0003	7.03
hcmv-miR-US33-3p	12.43 ± 1.63	83.83 ± 29.92	0.0003	6.74
**Validation set**	**(n = 68)**	**(n = 65)**		
hcmv-miR-US4-3p	30.35 ± 4.19	153.7 ± 49.20	0.0007	5.06
hcmv-miR-US29-3p	9.32 ± 1.38	43.00 ± 10.96	0.0009	4.61
hcmv-miR-US5-2-3p	6.53 ± 1.32	18.75 ± 4.22	0.0037	2.87
hcmv-miR-UL112-3p	180.8 ± 23.21	741.7 ± 205.8	0.0012	4.1
hcmv-miR-US33-3p	21.09 ± 3.32	80.88 ± 26.96	0.0086	3.83

a The relative expression of miRNA to spiked-in exogenous MIR2911(×10^-5^) was calculated using the 2^−ΔCq^ method. The data are presented as the mean ± SEM. P-value was calculated with a nonparametric Mann-Whitney test. The bold values represent the sample size of control subjects and fever patients in each set.

We examined the five selected HCMV miRNAs in a larger cohort comprising 66 fever patients and 68 controls (referred to as the validation set). The RT-qPCR results showed the alteration pattern of the five candidate miRNAs in the fever case group was consistent with that from the training set (*P* < 0.01 for all miRNAs) (**Table 2**), thus confirming the stability of the expression profile.

### 3.3 Confirmation of altered HCMV miRNAs in the serum samples of the testing set

**Figure 2:**
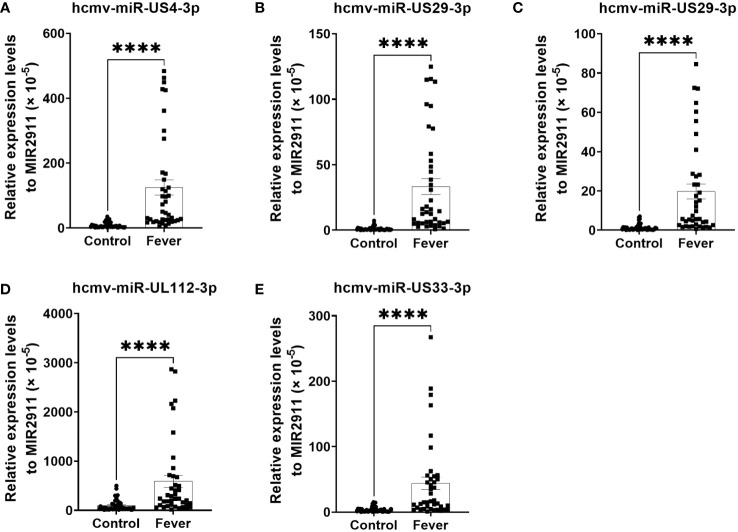
Relative levels of the five HCMV miRNAs in the serum of fever patients and healthy controls of the testing set **(A–E)**. Cq values were converted to relative concentrations normalized to MIR2911 values (× 10^-5^) and were calculated using the comparative Cq method (2^-ΔCq^). P values were calculated by the Mann-Whitney U test. ****P < 0.0001.

The changes in the five selected HCMV miRNAs expression were further confirmed in a cohort referred to as the testing set of 40 fever patients and 45 controls. As shown in **Figure 2**, the levels of the five selected HCMV miRNAs in the testing set were significantly elevated in the fever patients in comparison with the healthy controls (all with P < 0.0001), consistent with the results of the training and validation sets.

### 3.4 ROC curve analysis

**Figure 3:**
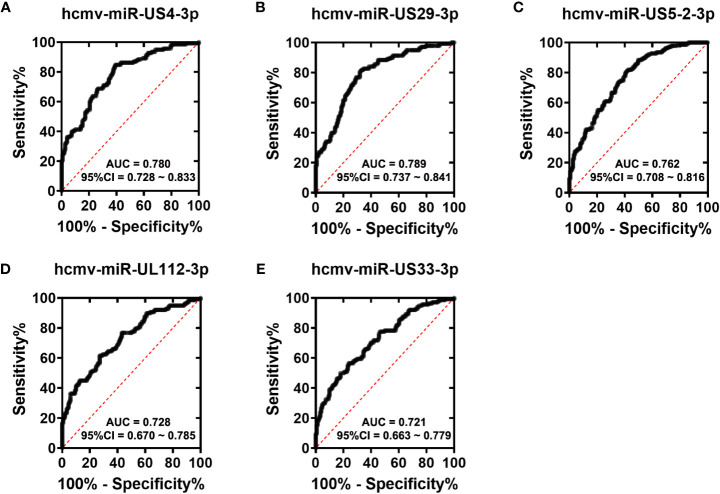
ROC curves analysis of the five selected HCMV miRNAs **(A–E)**. Receiver operating characteristic (ROC) curves for the ability of five individual HCMV miRNAs to discriminating fever patients from controls subjects in all the samples that were enrolled in the training set, validation set and the testing sets.

To assess the diagnostic value of the five HCMV miRNAs in discriminating fever patients from control subjects, we conducted the ROC curve analysis in the all samples enrolled in the training, validation, and testing sets. As shown in **Figure 3**, the area under the ROC curves (AUCs) of the five HCMV miRNAs for discriminating fever patients from controls ranged from 0.721–0.789, indicating the practical usefulness of individual HCMV miRNAs in the auxiliary diagnosis of the fever patients.

### 3.5 Association between serum HCMV miRNA expression levels and fever status

We then tested the predictive effects of the five HCMV miRNAs for fever among all patients and controls enrolled in the training, validation, and testing sets. Using the control group as the reference category and fever status as a dependent two-category variable, forward stepwise univariate logistic regression analysis demonstrated the five selected miRNAs were independent risk factors for FUO. The odds ratios (ORs) of these miRNAs for FUO were hcmv-miR-US4-3p: OR = 8.688, 95% CI, 4.923–15.331, *P* < 0.001, hcmv-miR-US29-3p: OR = 9.13, 95% CI, 5.268–15.826, *P* < 0.001, hcmv-miR-US5-2-3p: OR = 6.144, 95% CI, 3.580–10.546, *P* < 0.001, hcmv-miR-UL112-3p: OR = 4.303, 95% CI, 2.620–7.068, *P* < 0.001, and hcmv-miR-US33-3p: OR = 4.153, 95% CI, 2.510–6.870, *P* < 0.001 (**Table 3**).

**Table 3:** Univariate and multivariate logistic regression analyses of the five selected serum HCMV miRNAs as potential risk factors for patients with fever.

Variable	Univariate analysis			Multivariate analysis		
	OR	95% CI	P value	OR	95% CI	P value
**Age**	1.013	1.000-1.026	0.053	–	–	0.103
**Sex**	1.064	0.661-1.713	0.799	–	–	0.289
**hcmv-miR-US4-3p**	8.688	4.923-15.331	< 0.001	–	–	0.215
**hcmv-miR-US29-3p**	9.13	5.268-15.826	< 0.001	6.47	2.893-14.470	< 0.001
**hcmv-miR-US5-2-3p**	6.144	3.580-10.546	< 0.001	–	–	0.868
**hcmv-miR-UL112-3p**	4.303	2.620-7.068	< 0.001	–	–	0.517
**hcmv-miR-US33-3p**	4.153	2.510-6.870	< 0.001	2.753	1.255-6.043	0.012
**WBC**	1.257	1.147-1.377	< 0.001	1.311	1.123-1.530	0.001
**Lymphocyte**	0.904	0.881-0.928	< 0.001	0.928	0.899-0.957	< 0.001
**Monocytes**	1.347	1.205-1.506	< 0.001	1.617	1.346-1.942	< 0.001
**Neutrophil**	1.079	1.056-1.104	< 0.001	–	–	0.732
**Acidophilicgranulocyte**	0.839	0.737-0.955	0.008	0.797	0.685-0.927	0.003
**Basophil**	0.341	0.141-0.824	0.017	–	–	0.292

Next, we investigated whether the altered HCMV miRNAs were still independently associated with fever in the same cohort samples when age, gender, and other clinical parameters were included. Using a dependent two-category variable multivariate logistic regression analysis and after adjusting for age, gender, and hematological index, we observed that hcmv-miR-US29-3p: OR = 6.47, 95% CI, 2.893–14.470, *P* < 0.001 and hcmv-miR-US33-3p: OR = 3.2, 95% CI, 7.9–19.7, *P* < 0.001 remained independent risk factors in fever (**Table 3**).

Taken together, these results revealed that the five overexpressed miRNAs facilitate the diagnosis of fever and might serve as novel potential risk factors for fever.

### 3.6 Relative expression levels of the selected serum HCMV miRNA in fever patients with different etiology

We next explored the difference in the concentrations of the 5 HCMV miRNA among fever patients that were caused by different etiology. As shown in the Supplementary **Figure 1**, none of the five HCMV-encoded miRNAs has any statistically difference among patients with different etiology by Kruskal-Wallis’s test (Supplementary **Figure 1**). These results indicated that the altered HCMV miRNAs in fever patients may not affect by different etiology, but may associate with immune disorders.

### 3.7 Detection of HCMV in the peripheral blood and serum samples

**Figure 4:**
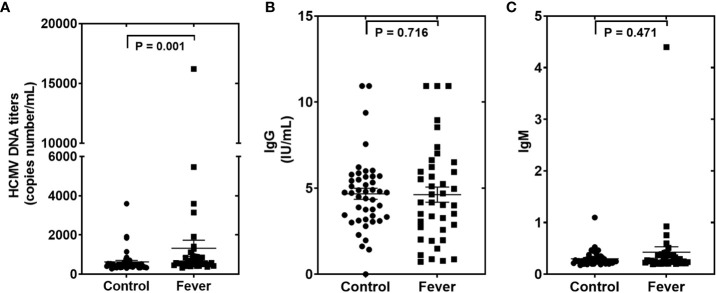
The human cytomegalovirus (HCMV) DNA, anti-HCMV IgG, and anti-HCMV IgM levels in patients with fever and healthy controls of the testing set **(A–C)**. Comparison of HCMV DNA copy numbers. **(A)**, anti-HCMV IgG **(B)**, and anti-HCMV IgM **(C)** in the fever patient’s vs the control subjects. P values were calculated by the Mann-Whitney U test.

We then measured the HCMV DNA in peripheral blood leukocytes (PBLs) of the samples in the testing set using RT-qPCR assays. Using the TaqMan assay, we found that the HCMV DNA was significantly higher in the fever patients than in the controls (**Figure 4**). Furthermore, we also performed ELISA assays to determine the anti-HCMV IgG and IgM concentrations in the serum samples of the same cohort (**Figure 4**). Our results showed that 100% of fever patients and 97.8% of controls were anti-HCMV IgG positive, while 97.5% of fever patients and 97.8% of controls were anti-HCMV IgM negative. No significant differences were found between the fever patients and the controls (**Figure 4**).

### 3.8 Correlations between the concentration of serum HCMV miRNAs and hematological parameters

To further examine the clinical association between the five selected HCMV miRNAs and hematological indices, we conducted a multiple nonparametric correlation analysis. The results showed that the concentrations of four of the five HCMV miRNAs were significantly negatively correlated with the blood C-reaction protein (CRP) concentrations in all fever patients (standardized coefficient R ranged from −0.151 to −0.217; *P* < 0.05) (**Table 4**). These data indicate that increased HCMV virus titers or virus reactivation were correlated with the severity of the immune response of fever patients. Nevertheless, no significant association was observed between the concentrations of the candidate miRNAs and other blood parameters (**Table 4**).

**Table 4:** Relationship between the five selected HCMV miRNAs expression levels in serum and hematological and serological indices^a^.

miRNA		WBC	Lymphocyte	Monocytes	Neutrophil	Acidophilicgranulocyte	Basophil	CRP	Globularproteins	PCT	IL-6
**hcmv-miR-US4-3p**	*R*	-0.091	0.046	-0.089	-0.026	-0.066	-0.024	-0.151	-0.077	0.174	-0.209
	*P value*	0.291	0.591	0.299	0.765	0.443	0.781	0.084	0.377	0.131	0.116
**hcmv-miR-US29-3p**	*R*	-0.116	0.047	-0.032	-0.039	-0.017	-0.021	-0.199	-0.146	0.143	-0.220
	*P value*	0.174	0.588	0.713	0.654	0.846	0.807	0.023	0.091	0.215	0.098
**hcmv-miR-US-5-2-3p**	*R*	-0.098	0.053	-0.027	-0.047	-0.007	0.021	-0.217	-0.113	0.183	-0.186
	*P value*	0.255	0.539	0.751	0.588	0.940	0.803	0.013	0.194	0.112	0.161
**hcmv-miR-UL112-3p**	*R*	-0.046	0.027	-0.024	-0.031	0.008	0.007	-0.184	-0.082	0.137	-0.178
	*P value*	0.591	0.758	0.778	0.721	0.926	0.932	0.036	0.346	0.234	0.180
**hcmv-miR-US33-3p**	*R*	-0.071	0.064	-0.063	-0.052	-0.040	0.025	-0.181	-0.070	0.065	-0.159
	*P value*	0.411	0.457	0.463	0.550	0.645	0.776	0.039	0.417	0.572	0.234

a Spearman rank correlation analysis.

**Figure 5:**
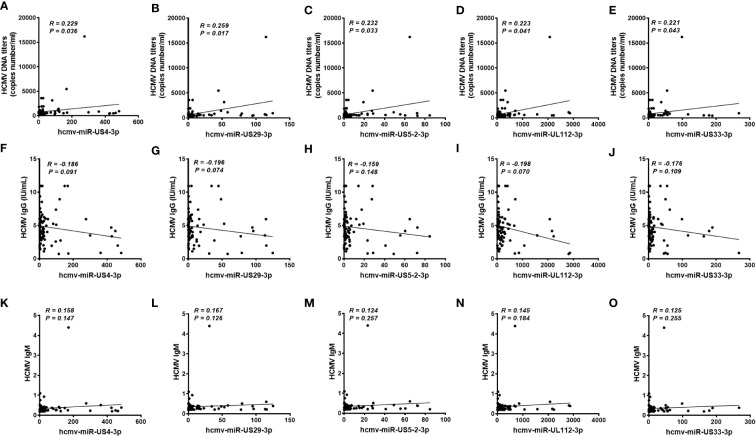
Correlation analysis of serum levels of the five selected HCMV miRNAs and the human cytomegalovirus (HCMV) DNA, anti-HCMV IgG, and anti-HCMV IgM levels in patients with fever and healthy controls of the testing set **(A–O)**. Correlations between the serum levels of the five selected HCMV miRNAs and HCMV DNA contents **(A-E)**, anti-HCMV IgG **(F-J)** and anti-HCMV IgM **(K-O)**.

We also analyzed the association between the concentrations of the five serum HCMV miRNAs and the contents of HCMV DNA titers and serum HCMV IgG/IgM in the testing set samples. As expected, there was no significant statistical correlation between HCMV miRNAs and HCMV IgG/IgM indicators (*P* > 0.05) (**Figure 5**). Notably, the serum levels of the five HCMV miRNA were significantly correlated with the contents of HCMV DNA titers (*P* < 0.05) (**Figure 5**).

### 3.9 Target gene analysis of HCMV miRNA

To explore the potential biological function of the five HCMV miRNAs, target genes prediction analyzes for the five HCMV miRNAs were performed by using TargetScan Human 5.2 Custom (https://www.targetscan.org/vert_50/seedmatch.html). As showed in **Supplementary Table 2**, a total of 143, 10, 117, 33, and 7 genes were predicted as potential target of hcmv-miR-US4-3p, hcmv-miR-US29-3p, hcmv-miR-US5-2-3p, hcmv-miR-UL112-3p and hcmv-miR-US33-3p, respectively. Notably, five genes including NUFIP2, ANKH, TNPO1, BCL11A, and LRRC59 were the common targets of hcmv-miR-US4-3p, hcmv-miR-UL112-3p and hcmv-miR-US5-2-3p.

## 4 Discussion

Despite the rapid progress in the comprehensive physical examination and exhaustive workup in the hospital, fever remains a diagnostic and therapeutic challenge for expert physicians, especially in primary treatment (22). This is because the etiologies of fever vary with the region and time. Infections and NIID are the leading cause of fever. However, reportedly, the ratios of undiagnosed fever cases have gradually increased (4, 23). This led us to explore the potential etiology and diagnostic methods for fever. HCMV is a highly prevalent virus in the human population and an important infectious pathogen that induces morbidity and mortality in immunocompromised individuals. Unfortunately, the mechanism of HCMV pathogenesis remains obscure (10). Patients with fever usually have significantly immunocompromised signs, which can be induced by many pathogens (24). Many studies have demonstrated that miRNAs—small non-coding RNAs—are involved in HCMV pathogenesis. These miRNAs alter the host immune system. However, the clinical relevance between HCMV-encoded miRNAs and fever has rarely been investigated. We designed a retrospective case-control study to uncover the potential association between HCMV and fever. By conducting a comprehensive profiling analysis of HCMV miRNAs in the serum of fever patients using RT-qPCR, we found a higher prevalence of HCMV miRNAs in fever patients, and 17 out of 24 (70.8%) measured HCMV miRNAs were markedly elevated in serum samples from fever patients compared with the normal controls. Moreover, ROC curves and multiple rank correlation analyses revealed a strong relationship between selected HCMV miRNAs and fever status. To date, this study is the first comprehensive analysis of circulating HCMV miRNA in fever patients. Our results not only reveal an increased number of novel risk factors for molecular assessment and management of fever but also provide potential etiology, pathogenesis, and progression mechanism of HCMV.

HCMV reactivates in response to immunosuppression, inflammation, infection, or stress (10). Evidence shows the potential link between HCMV infection and fever (25); however, the pathologic function of HCMV involved in these diseases remains relatively unexplored (26). A prospective multicenter survey of 154 patients with fever in twelve Turkish tertiary care hospitals found five cases diagnosed as CMV infection (23). Another group found that HCMV reactivation was related to fever patients during neutropenia (27). HCMV was also detected in the colonic mucosa of patients with fever (28). As the second most common cause of fever, the pathologic mechanisms of CTD are still largely unknown, and HCMV infection has been reported to participate in CTD pathogenesis (29, 30). Studies have observed a higher prevalence or activity of HCMV in various autoimmune diseases, such as systemic lupus erythematosus (31) and systemic sclerosis (32). Collectively, these data further highlight the clinical relevance of HCMV and fever. We found that the levels of most HCMV miRNAs were increased in the blood of fever patients, elucidating a potential link between HCMV and fever. The univariate logistic regression analyses revealed that the five HCMV miRNAs were independently correlated with fever using the control group as the reference category. Moreover, after multivariate logistic regression with adjustment for age, gender, and other hematological indices, HCMV-encoded miR-US29-3p and miR-US33-3p remained a strong risk factor for fever. Additionally, four of the five miRNAs were significantly associated with CRP levels. The exact reason for the negative correlation between the serum HCMV miRNAs’ levels and blood CRP contents remain unclear. One possible explanation is that HCMV under latent infection status in healthy subjects with normal immunity, however, fever patients with high CRP levels often exhibited immunocompromised status, and the HCMV can be reactive when the host organism immunity attenuate (33). Nevertheless, Further studies and direct evidences are still needed to clarify this issue. Thus, these results indicated that HCMV miRNAs might act as a novel auxiliary evaluation index for fever and are closely associated with immune response. Our findings contribute to the early implementation of medical treatment and provide useful information to help physicians refine their early therapeutic interventions.

The intrinsic mechanisms of HCMV miRNA upregulation in the circulation of fever patients are yet to be elucidated. Multiple studies from our group and others have documented that miRNAs are either selectively packed into microvesicles and actively secreted or they passively leak from broken cells into the circulation (34). Moreover, HCMV can deliver its miRNAs through virions and dense bodies (35, 36). We speculated that activated HCMV in the infected cells might release miRNAs into the blood. However, we did not study the state of HCMV in the present study. Emerging studies have reported the presence of cell-free viral miRNAs in the plasma derived from the active release of viral particles or lysis of infected cells. The viral miRNAs were detectable in systemically circulating exosomes in EBV and KSHV-associated malignancies patients (37, 38). Moreover, human JC polyomavirus-encoded miRNAs can be detected in the plasma and urine samples of progressive multifocal leukoencephalopathy patients (39). These studies further confirm our hypothesis. Although the cell types releasing HCMV miRNAs into the blood remain unclear, HCMV might target the highly susceptible cell types, such as endothelial cells, macrophages, and monocytes. However, further studies need to confirm the exact origin of these miRNAs.

Our study confirmed five of the 17 upregulated HCMV miRNAs to be significantly elevated in the serum of fever patients. Of the five altered miRNAs, miR-UL112-3p was the most reported miRNA. It is ectopically expressed early after infection and accumulates during viral infection. Many studies suggest that this miRNA might be involved in regulating both host and viral gene expression during viral infection to establish and maintain latency through targeting genes, such as MICB, immediate-early (IE) gene products, Toll-Like Receptor (TLR), and Interleukin-32 (IL-32) (11). Remarkably, two recent studies highlighted that serum levels of miR-UL112-3p were significantly elevated in patients with essential hypertension, diabetes mellitus, and glioblastoma (14, 15). In addition, immune and inflammation-associated interferon regulatory factor 1 was demonstrated to be a direct target of miR-UL112-3p (14). Abundant expression of miR-US5-2-3p was observed during HCMV lytic infection in human embryo lung fibroblasts, suggesting that these miRNAs function in the HCMV replication cycle and viral DNA synthesis (40). miR-US5-2-3p was also reported to regulate the viral gene US7 later in the infection (41). A recent study found that the two HCMV miRNAs identified in our study can cooperatively target multiple genes belonging to the cell secretory pathway to limit cytokine release and aid in the proper assembly and release of the viral particles (42). Currently, we are unaware of the role of miR-US4-3p, miR-US29-3p, and miR-US33-3p. Our results and the abovementioned findings suggest that the HCMV miRNAs identified in our study might be involved in the pathological process of fever and immune disorders by regulating HCMV infection-related genes. This idea will be further validated in ongoing *in vitro* and *in vivo* studies.

Our bioinformatic analysis results revealed that a number of genes could be regulated by the five HCMV miRNAs. More interestingly, of these predicted targets, five genes including NUFIP2, ANKH, TNPO1, BCL11A, and LRRC59 were recognized as the common targets of hcmv-miR-US4-3p, hcmv-miR-UL112-3p and hcmv-miR-US5-2-3p. Performing the literature survey for the five genes, NUFIP2 were found to be closely associated with immune disease, including rheumatoid arthritis, and may participate in immune response-regulating signaling pathway as well as immune cell function (43, 44). Like NUFIP2, ANKH was also reported to be involved in the pathogenesis of arthritis, immune and inflammatory response (45, 46). For TNPO1, reports found that this gene was associated with protein localization and transport, inflammatory-immune response, and tumor immune evasion (47–49). BCL11A was reported to be associated with adaptive and innate immunity, especially for dendritic cell and lymphoid development, and genome-wide association studies demonstrated that this gene is participated in immune-mediated inflammatory disease such as COVID-19 and autoimmune diseases (50–53). LRRC59 was identified as a vital positive regulator of type I IFN signaling upon virus infection and acted role in innate antiviral responses (54). Moreover, upregulated LRRC59 protein levels were reported after immune stimulation and would greatly facilitate the processes of lymphocytes immune phenotypes occurring (55). Collectively, these evidences further indicated that the altered HCMV miRNAs are therefore likely to be play pivot roles in inflammatory-immune disorders.

Our study has some limitations. First, we observed no significant difference in the levels of sera IgG and IgM in fever patients when compared with controls, and these results revealed no direct evidence for CMV activation in those fever patients. The high positive rate for IgG and high negative rate for IgM were also reported in other similar pioneer studies (14, 15). These studies and our findings suggest that the detected IgG/IgM may not reflect the actual virus activation in the HCMV-infected and reactivation population. Nevertheless, HCMV DNA titers were elevated in the serum samples of fever patients compared with the controls and were positively correlated with the levels of the five HCMV miRNAs. These results can partially prove that HCMV reactivation occurs in fever patients with immune disorders. Another weak point of the present study is that all the fever patients and controls in our study were Chinese Han from one medical center. Since the causes of fever vary depending on the region and time, we cannot assume that the HCMV miRNA profiles will be consistent in other ethnicities and medical centers.

In conclusion, our study demonstrated for the first time that HCMV miRNAs can be detected in the circulation of fever patients and altered HCMV miRNAs are common in fever patients with immune disorders. Moreover, altered HCMV miRNAs could be novel risk factors for fever, allowing further auxiliary diagnosis and management of fever patients. Our data reveal a novel phenomenon that HCMV miRNA expression patterns might reflect the immune response of the host, and provide new clues and direction to explore the etiology of fever.

## Data availability statement

The original contributions presented in the study are included in the article/**Supplementary Material**. Further inquiries can be directed to the corresponding authors.

## Ethics statement

The studies involving human participants were reviewed and approved by the ethics committee board of Jinling hospital. Written informed consent to participate in this study was provided by the participants’ legal guardian/next of kin.

## Author contributions

Conceptualization, C-YZ, CNZ, and J-JW. Data curation, ChengW, MD, YZ, PC, and CPZ. Formal analysis, ChengW, WZ, ChenW, and JW. Funding acquisition, ChengW and CNZ. Methodology, ChengW, MD, XC, CNZ, and C-YZ. Resources, J-JW, C-YZ, YZ, and CNZ. Supervision, CNZ, C-YZ, and J-JW. Roles/writing and original draft, ChengW and CNZ. Writing, review and editing, J-JW, ChenW, and C-YZ. All authors contributed to the article and approved the submitted version.
