# Supplementary material for: Altered serum human cytomegalovirus microRNA levels are common and closely associated with the inflammatory status in patients with fever
Source: Front Immunol. 2022 Dec 14;13:1079259. doi: 10.3389/fimmu.2022.1079259 (PMC9795028; doi:10.3389/fimmu.2022.1079259)
Supplement: Supplementary file 1 [file DataSheet_1.zip › Data Sheet 1/Supplementary Data Table and Figure.doc]

**Altered serum human cytomegalovirus microRNA levels are common and closely associated with the inflammatory status in patients with fever**

Cheng Wang^1,2,†^, Yunhua Zhu^1,2,†^, Penglu Chen^1,2,†^, Chen Wang^2^, Wanqing Zhou^1,2^, Cuiping Zhang^1^, Jing Wang^1^, Xi Chen^2^, Meng Ding^1,2,*^, Chunni Zhang^1,2,*^ Jun-Jun Wang^1,*^, Chen-Yu Zhang^1,2,*^

^1^ Department of Clinical Laboratory, Jinling Hospital, State Key Laboratory of Pharmaceutical Biotechnology, NJU Advanced Institute for Life Sciences (NAILS), School of Life Sciences, Nanjing University, Nanjing, China;

^2^ Nanjing Drum Tower Hospital Center of Molecular Diagnostic and Therapy, Chinese Academy of Medical Sciences Research Unit of Extracellular RNA, State Key Laboratory of Pharmaceutical Biotechnology, Jiangsu Engineering Research Center for MicroRNA Biology and Biotechnology, NJU Advanced Institute of Life Sciences (NAILS), Institute of Artificial Intelligence Biomedicine, School of Life Sciences, Nanjing University, Nanjing, China;

*** Correspondence:** Chen-Yu Zhang at State Key Laboratory of Pharmaceutical Biotechnology, Nanjing Advanced Institute for Life Sciences (NAILS), School of Life Sciences, Nanjing University, 163 Xianlin Road, Nanjing 210046, China. E-mail cyzhang@nju.edu.cn. Meng Ding, Chunni Zhang and/or Junjun Wang at Department of Clinical Laboratory, Jinling Hospital, 305 East Zhongshan Road, Nanjing, 210002, China. E-mail vikkiding@163.com, zchunni27@hotmail.com and/or wangjunjun9202@163.com.

^†^These authors contributed equally to this work.

**Running title:** Serum HCMV miRNAs in fever

**Keywords:** human cytomegalovirus; serum; microRNA; fever; risk factor; immune disorders

**Supplementary Table and Figure**

**Supplementary Table 1.** Expression profile of HCMV-encoded miRNAs in fever patients and healthy controls in the training set.

| **HCMV miRNA** | **Control** | **Fever** | **P value** | **Fold change** |
| --- | --- | --- | --- | --- |
| hcmv-miR-US4-3p | 29.87 ± 5.55 | 209.7 ± 61.02 | < 0.0001 | 7.02 |
| hcmv-miR-US4-5p | 21.64 ± 4.84 | 104.6 ± 31.19 | 0.0002 | 4.83 |
| hcmv-miR-US29-3p | 6.56 ± 1.52 | 34.12 ± 9.72 | < 0.0001 | 5.2 |
| hcmv-miR-UL70-5p | 47.23 ± 7.61 | 232.6 ± 88.49 | 0.0037 | 4.92 |
| hcmv-miR-US5-2-3p | 2.74 ± 0.60 | 14.17 ± 4.07 | 0.0001 | 5.17 |
| hcmv-miR-UL112-3p | 123.3 ± 15.92 | 867.7 ± 312.0 | 0.0003 | 7.03 |
| hcmv-miR-UL148D | 1128 ± 278.3 | 4192 ± 1443 | 0.0014 | 3.72 |
| hcmv-miR-US25-2-3p | 106.4 ± 14.85 | 369.5 ± 91.47 | 0.0017 | 3.47 |
| hcmv-miR-US33-3p | 12.43 ± 1.63 | 83.83 ± 29.92 | 0.0003 | 6.74 |
| hcmv-miR-US33-5p | 50.66 ± 21.30 | 136.8 ± 45.90 | 0.037 | 2.7 |
| hcmv-miR-US5-2-5p | 823.9 ± 562.3 | 633.9 ± 184.8 | 0.0057 | 0.77 |
| hcmv-miR-UL69 | 3.05 ± 1.07 | 6.47 ± 3.76 | 0.341 | 2.12 |
| hcmv-miR-US25-2-5p | 126.9 ± 19.24 | 254.3 ± 97.4 | 0.314 | 2.004 |
| hcmv-miR-UL59 | 123.5 ± 28.90 | 198.8 ± 72.01 | 0.902 | 1.61 |
| hcmv-miR-US5-1 | 40.82 ± 4.22 | 175.2 ± 69.38 | 0.155 | 4.29 |
| hcmv-miR-US22-5p | 348.9 ± 42.76 | 2720 ± 973.6 | 0.0017 | 7.8 |
| hcmv-miR-US22-3p | 11.88 ± 1.69 | 70.05 ± 24.69 | 0.0004 | 5.9 |
| hcmv-miR-UL36-3p | 20.11 ± 2.92 | 124.3 ± 42.08 | 0.0035 | 6.18 |
| hcmv-miR-UL36-5p | 111.1 ± 29.96 | 393.8 ± 109.7 | 0.0026 | 3.54 |
| hcmv-miR-UL112-5p | 18.66 ± 6.14 | 101.5 ± 37.11 | 0.0038 | 5.44 |
| hcmv-miR-US25-1-5p | 2.58 ± 0.41 | 2.70 ± 0.29 | 0.626 | 1.05 |
| hcmv-miR-US25-1-3p | 674.8 ± 175.6 | 1455 ± 386.0 | 0.075 | 2.16 |
| hcmv-miR-UL22a-5p | 33.61 ± 5.43 | 158.3 ± 51.33 | 0.0065 | 4.71 |
| hcmv-miR-UL22a-3p | 13.53 ± 1.56 | 17.91 ± 1.98 | 0.193 | 1.32 |

**Supplementary Figure 1.** **Relative expression levels of the five HCMV miRNAs in the fever patients with different etiology (A–E).** Cq values were converted to relative concentrations normalized to MIR2911 values (× 10^-5^) and were calculated using the comparative Cq method (2^-ΔCq^). P values were calculated by the Kruskal-Wallis’s test.
